# Radiofrequency ablation for the treatment of haemorrhoidal disease: a minimally invasive and effective treatment modality

**DOI:** 10.1007/s10151-019-02054-2

**Published:** 2019-08-09

**Authors:** M. M. R. Eddama, M. Everson, S. Renshaw, T. Taj, R. Boulton, J. Crosbie, C. Richard Cohen

**Affiliations:** 1 Department of Colorectal Surgery, University College London Hospital, London, UK; 2 Division of Surgery and Interventional Science, University College London, London, UK; 3 GI Services, 250 Euston Road, London, NW1 2PG UK

**Keywords:** Patient reported outcome, Hemorrhoids, Radiofrequency ablation, Minimally invasive surgical procedures

## Abstract

**Background:**

Haemorrhoidal disease (HD) is a common colorectal condition that often requires surgical treatment. Less invasive procedures are usually more acceptable to patients. The aim of this study was to report the outcome of a novel and minimally invasive technique employing a radiofrequency ablation (RFA) energy (Rafaelo^®^) to treat HD.

**Methods:**

A total number of 27 patients who had RFA for the treatment of HD were recruited to this study. The procedure was performed under deep sedation and local anaesthesia. Patients’ demographics; haemorrhoid severity score (HSS); quality of life; pain and satisfaction scores; and recurrence rate were recorded.

**Results:**

The mean age of the patients was 46 (SD 14) years, 18 (67%) males and 9 (33%) females. The mean body mass index was 25 (SD 4) kg/m^2^. The predominant symptom of all patients was per-rectal bleeding. HSS improved from 7.2 (SD 1.9) before the procedure to 1.6 (SD 1) after the procedure (*p* < 0.0001). Postoperative pain scores on a scale of 0–10 were 0, 2 (SD 2), 1 (SD 2), and 0 on immediate, day-1, day-3, and 2-month follow-up questionnaire. The mean satisfacion score was 9 (SD 1.5) out of 10 on 2-month follow-up. Mean time until patients returned to normal daily activity was 3 (SD 1) days following the procedure. Quality-of-life assessments including: visual analogue scale scores (before: mean 70, SD 23; after: mean 82, SD 16; *p* < 0.001) and EQ-5D-5L (before: mean 0.84, SD 0.15; after: mean 0.94, SD 0.13; *p* < 0.05) were significantly improved. The mean length of follow-up for recurrence of symptoms was 20 months (range 12–32 months). One patient (4%) reported the recurrence of rectal bleeding 12 months after the procedure.

**Conclusions:**

RFA for the treatment of HD is safe and effective in achieving symptomatic relief. It is associated with minimal postoperative pain and low incidence of recurrence.

## Introduction

Pathological enlargement and displacement of the anal vascular cushions referred to as haemorrhoidal disease (HD) may be associated with pain, itching, bleeding, discharge (soiling), and haemorrhoidal tissue prolapse [1]. Although the true prevalence is unknown due to many patients being too embarrassed to seek treatment, a significant number of people around the world pursue medical and surgical treatment. Thus, HD is both a medical problem and a socioeconomic burden [2].

In patients who have persisting symptoms despite conservative treatment, there are several surgical options available. While excisional haemorrhoidectomy is considered to be the most effective treatment, significant postoperative pain and changes in the anatomy of the anal canal can impair defecation. This has led surgeons to adopt more tolerable procedures, such as stapled haemorrhoidectomy, haemorrhoidal artery ligation, haemorrhoidal dearterialisation, rubber banding ligation, infrared coagulation, and sclerotherapy or phenol injection. Yet, no single technique has been universally accepted as the best treatment [3]. The therapeutic choice of treatment is largely dependent on the severity of the symptoms, size of haemorrhoidal tissue, and extent of displacement. For example, HD with severe prolapsing skin that makes it difficult for the patient to maintain hygiene may require excision. However, patients’ preference tends to gravitate towards less invasive procedures.

Radiofrequency ablation (RFA) utilises an electrical current produced by radio waves to generate heat. For many years, this method has been used to ablate dysfunctional tissue in benign and malignant disease [4]. Akin to sclerotherapy, rubber banding, and infrared coagulation, RFA for early stage HD has shown promising results [5]. It causes obliteration of vascular channels, fibrosis, and shrinkage of the anorectal cushions. Long term, these changes can lead to plication of anorectal mucosa and alleviation of symptoms. The effect of RFA on tissue has been reported previously in vein and tumour ablation [6]. Histologically, tissues demonstrated significant uniform collagen contraction and fibrosis [7, 8]. For the first time, RFA for HD has been offered to National Health Service (NHS) patients in the United Kingdom by University College London Hospital NHS trust (UCLH). The aim of the present study was to evaluate the outcomes of RFA for the treatment of HD.

## Materials and methods

A total number of 27 patients had RFA for HD and were included in a pilot study at UCLH, between the period February 2016 and October 2018. The procedure was approved by the Trust’s Clinical Effectiveness Steering Committee. Data collection and analysis was approved as service evaluation by our audit department. Recruitment of patients was conducted in the colorectal outpatient clinic. Inclusion criteria were as follows: patients with symptomatic HD; grade II–IV HD; age between 18 and 80 years; and ability to give written consent for the procedure and the study. Those who required concomitant interventions for other perianal conditions, such as anal fissure or skin tag excision, were excluded from the analysis. Patients were warned about the possibility of surgical bleeding, failure and pain post operatively as with all haemorrhoidal procedures.

Face-to-face and telephone interviews were conducted pre- and postoperatively by the direct care team to collect data on patient demographics; diagnostics; postoperative pain and satisfaction scores; and procedure-related complications. We also gave patients a questionnaire to assess their Haemorrhoid Severity Score (HSS), including pain, itching, discomfort, bleeding, soiling, and haemorrhoidal tissue prolapse [9, 10] (Table 1); anal incontinence and quality of life 2–3 weeks before and 6–8 weeks after the procedure. Cleveland Incontinence Score (CIS), European Quality-of-Life Five Dimension, Five Level (EQ-5D-5L), and Visual Analogue Scale (VAS) scores were assessed before and 2 months after the procedure. Patients were asked to rate their quality of life in relation to suffering from haemorrhoidal symptoms on a VAS ranging from 0 to 100, (0 being worst and 100 being the best quality of life).

**Table 1:** Haemorrhoid severity score questionnaire

Question	0	1	2	3
How often do you have pain from the haemorrhoid?	□	□	□	□
How often do you have itching or discomfort of the anus?	□	□	□	□
How often do you have bleeding when passing motion?	□	□	□	□
How often do you soil your underclothes (soiling from the anus)?	□	□	□	□
How often do you reduce a prolapsing haemorrhoid with your hand when passing a motion?	□	□	□	□

The score was graded as: 0, never; 1, less than once a week; 2, 1–6 times weekly; and 3, every day (always). The maximum score was 15 points. Statistical analysis was performed using paired *t* test before and after the procedure

CIS is widely accepted because it fulfills the criteria of being simple and accurate. It permits objective comparison of levels of incontinence among patients. It assesses incontinence for solids, liquid, gas, and also whether the patients wear pads or suffer alterations to their quality of life as a result of their anal incontinence [11].

VAS is a scale, where patients are asked to indicate by drawing a line of their overall health at the time of assessment. It usually offers complementary information to EQ-5D-5L [12]. Furthermore, EQ-5D-5L reports information on five dimensions of patient’s life including: mobility; self-care; usual activities; pain/discomfort; and anxiety/depression.

### Surgical technique

**Fig. 1:**
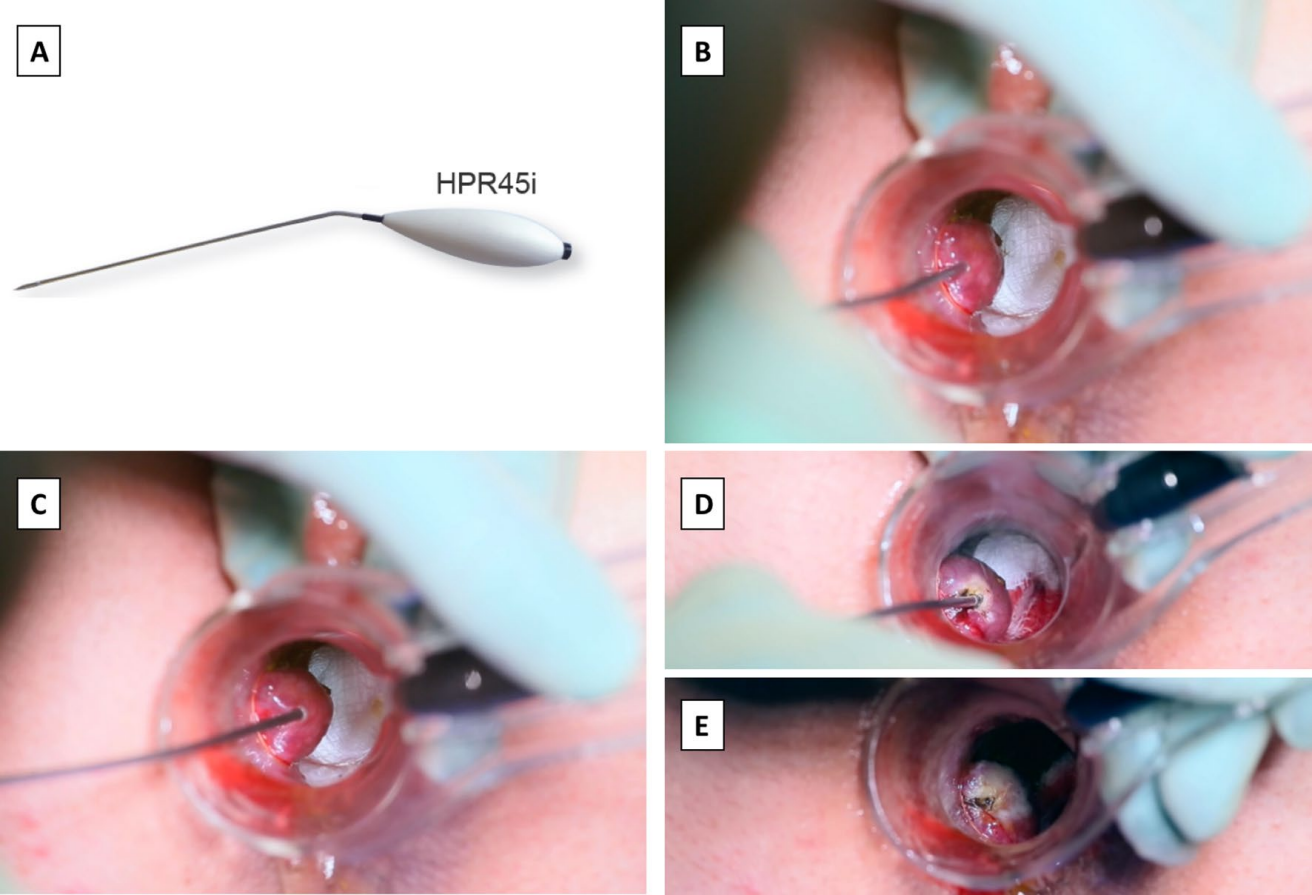
HPR45i probe (**a**) is inserted into the haemorrhoidal tissue (**b**), tissue is tilted away from the submucosa (**c**), and the radiofrequency energy is applied until the tissue exhibits whitish discoloration (**d**, **e**)

The procedure was performed under deep sedation, or light general anaesthesia with the patient positioned in lithotomy. Perianal block was performed using approximately 40 ml of bupivacaine 0.25%. We used a 69 × 23 mm proctoscope with 100 mm-long handle and a high intensity LED light source (Fcaresystems, Antwerpen, Belgium). The proctoscope has a simple vent on one side, through which a single haemorrhoidal tissue protrudes, while the rest of the anorectal cushion is compressed. At a level approximately 5 mm above the dentate line, the submucosa of haemorrhoidal tissue was infiltrated with approximately 1 ml of bupivacaine 0.25%. In addition to achieving local anaesthesia, this step aimed to create a fluid barrier and subsequently prevent the transmission of heat to the internal anal sphincter muscle. The Rafaelo^®^ device and associated HPR45i probe (Fcaresystems, Antwerpen, Belgium) were used to deploy RFA energy of 4 MHz frequency to the haemorrhoidal tissue. The tip of the probe was inserted fully into the haemorrhoid tissue approximately to a depth of 5–10 mm, at an approximately 30° angle to the tissue surface (Fig. 1a, b). The haemorrhoidal tissue was tilted away from the submucosal layer (Fig. 1c). The application of RFA was continued until the tissue exhibited whitish discolouration, after which the energy was applied to the external surface of the haemorrhoidal tissue to optimise tissue desiccation (Fig. 1d, e). A maximum of 3000 MHz with a power sitting of 25 W was applied to an individual haemorrhoidal tissue at one time. A cold saline soaked tonsillar swab was immediately applied to the surface of the haemorrhoidal tissue. Any bleeding was controlled by inducing coagulation using the radiofrequency probe. Patients were discharged on routine analgesia including: paracetamol, codeine, and ibuprofen to take when required.

### Statistical analysis

Data were analysed using GraphPad Prism (GraphPad Prism version 6 for MAC OS X, GraphPad Software, San Diego California USA). For inference statistics, paired *t* test was used to analyse continuous data and Chi-square test was used to analyse categorical data. The level of statistical significance was set at 5% (*p* ≤ 0.05) for all test procedures. EQ-5D-5L Crosswalk Index Value Calculator was used to calculate the crosswalk index values for the EQ-5D-5L dimension scores (EQ-5D, Rotterdam Netherlands, http://www.euroqol.org).

## Results

Patient characteristics are summarised in Table 2.

**Table 2:** Patient characteristics (*n* = 27)

Age, years: mean (SD)	46 (14)
Sex	
Male	18 (67%)
Female	9 (33%)
BMI: mean (SD)	25 (4)
ASA score	
I	18 (67%)
II	9 (33%)
Smoker	
Yes	8 (30%)
No	19 (70%)
Racial origin	
African	3 (11%)
Asian	3 (11%)
Caucasian	14 (52%)
Middle Eastern	5 (19%)
Mixed	2 (7%)
Haemorrhoidal severity	
Grade 2	8 (30%)
Grade 3	10 (37%)
Grade 4	9 (33%)

*ASA* American Society of Anaesthesiologists, *BMI* body mass index, *SD* standard deviation

### Procedure

The number of haemorrhoids treated at one time was 1, 2, and 3 in 9 (33%), 15 (56%), and 3 (11%) patients, respectively. The median energy applied per haemorrhoid was 1025 J (range 50–3050 J). The median time spent per haemorrhoid treatment was 35 s (range 15–120 s). The median duration of the procedure was 10 min (range 5–19 min.

### Outcome

#### Haemorrhoid Severity Score

**Fig. 2:**
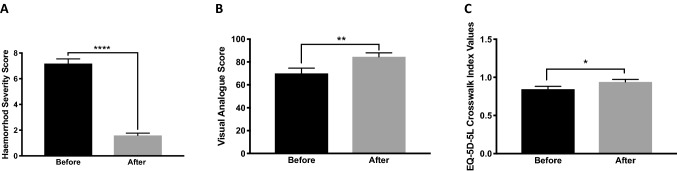
Haemorrhoidal disease severity and quality of life was assessed 2–3 weeks before and 6–8 weeks after the procedure. Haemorrhoidal Severity Score (**A**); Visual Analogue Score on a scale of 0–100 quality of life in relation to haemorrhoidal disease (**B**); and EQ-5D-5L crosswalk index values (**C**) were significantly improved comparison before and after the procedure. *p* < 0.05 (*). *p* < 0.01 (**). *p* < 0.0001 (****)

The severity of haemorrhoidal symptoms was assessed 2–3 weeks before and 6–8 weeks after the procedure. A score ranging from 0 to 15 was calculated. The mean score of HSS was improved from 7.2 (SD 1.9) before to 1.6 (SD 1) after the procedure (*p* < 0.0001) (Fig. 2A). The individual symptom scores and significance in difference before and after the procedure are summarised in Table 3.

**Table 3:** Haemorrhoid severity score, summary of individual symptom results before and after the procedure

Symptom^a^	Before: mean (SD)	After: mean (SD)	Significance
Pain	1.7 (0.6)	0.2 (0.1)	*p* < 0.0001
Itching	0.9 (0.6)	0.03 (0.1)	*p* < 0.0001
Bleeding	3 (0.2)	0.8 (0.4)	*p* < 0.0001
Soiling	0.2 (0.3)	0	*p* = 0.02
Haemorrhoidal tissue prolapse	1.3 (0.2)	0.5 (0.5)	*p* < 0.0001

Paired comparison was performed

^a^For each symptom, the minimum score was 0 (never) and the maximum score was 3 (always)

#### Postoperative pain

Postoperative pain scores (VAS 0–10) were 0, 2.4 (SD 2.3), 1.8 (SD 2.4), and 0 on immediate, day-1, day-3, and 2-month follow-up. Routine analgesia was used in 16 (60%), and 11 (40%) of the patients on postoperative days 1 and 2, respectively. None of the patients used analgesia in relation to their treated HD when reviewed at 8 weeks.

#### Patient satisfaction and fitness to return to normal daily activities

The mean satisfaction score was 9/10 (SD 1.5) at 2-month follow-up. The majority of patients (25, 93%) would recommend the procedure to other patients and found it reliable and effective. Although patients were given an official doctor’s notice to be off work for 10 days postoperatively, they reported that they felt able to return to their normal daily activities, including work, after a mean time of 3 days (SD 1).

#### Recurrence of symptoms

There was 1 patient who reported recurrence of per-rectal bleeding at 12-month follow-up. This patient originally had three haemorrhoids, all treated at the same time. In all other patients, a median follow-up period of 20 months (range 12–32 months) confirmed that there was no recurrence of symptoms, such as bleeding, pain, discharge, or itchiness. In terms of early complications, approximately 7 days following the initial procedure, 2 (8%) patients presented to the emergency department with per-rectal bleeding; this was minimal, and both were discharged from the emergency department with reassurance. There were no serious complications recorded.

#### Cleveland Incontinence Score

A total number of 18 patients recorded their CIS score immediately before and 2 months after the procedure. Three of them reported a score of 2, 3, and 4 before the procedure. Subsequently, these three patients reported a 0 CIS score after the procedure. A female patient reported a CIS score of 9 before and after the procedure. All other patients reported 0 CIS score before and after the procedure.

#### Quality-of-life assessment

A total number of 17 (63%) patients recorded their VAS and completed the EQ-5D-5L questionnaire immediately before and 2 months after the procedure. There was a significant (*p* < 0.001) improvement in the VAS score after the procedure (mean 82, SD 16) in comparison with before (mean 70, SD 23) (Fig. 2), 4 (23%) out of those who recorded their VAS score recorded a post procedure score of 100. Furthermore, EQ-5D-5L crosswalk index values were significantly higher (*p* < 0.05) after the procedure (mean 0.94, SD 0.13) in comparison with before (mean 0.84, SD 0.15).

## Discussion

Our main findings demonstrate that RFA for HD is associated with: minimal pain; improvement in HSS within 6–8 weeks of the procedure and patients’ quality of life; and an early return to work/normal activities. There were no serious complications nor evidence of damage to the anal sphincters. Recurrence after a mean follow-up of 20 months was observed in only 1 (4%) of the patients, in the form of recurrence of bleeding. These results are consistent with what was reported by Gupta and colleagues, who were the first to report patient outcomes after RFA for haemorrhoids [13, 14]. Gupta et al. also compared RFA to rubber band ligation for the treatment of grade 2 HD. RFA group was associated with less pain and a lower recurrence rate. However, the incidence of bleeding and prolapsing of haemorrhoids were comparatively higher [13]. Furthermore, the return to normal activities was significantly lower in the RFA group (mean 2 days) in comparison with rubber band ligation treatment (mean 5 days) [13]. Our cohort is different from those in the literature, because RFA was also offered to patients with grade 3 and 4 HD. Previously, RFA was only offered to patients with grade 1 and 2 HD [15]. In fact, 37% and 33% of our cohort suffered from grade 3 and 4 HD, respectively. Our rational for offering the procedure to patient with grade 3–4 haemorrhoids is to test its benefit for symptomatic relief of pain, itchiness, bleeding, or soiling. In particular, for grades 3 and 4 haemorrhoids, we did not expect the procedure to resolve chronic skin prolapse; however, RFA appears to be effective in resolving other symptoms. So far, based on our experience, we suggest that the procedure could be offered for all symptomatic HD as a first line. However, we would still advise caution and careful counselling of patients with mainly external haemorrhoids or substantial prolapsing haemorrhoids, where excision may be more effective.

Although we did not perform a cost-effective analysis, the estimated cost per patient was £300 British Pounds (GBP) for the HPR45i probe and the treatment packs (single use disposable). However, the Rafaelo^®^ EVRF machine (F Care Systems, Antwerpen, Belgium) was loaned to the hospital free of charge.

The strengths of this study may be the inclusion of all grades of HD; that we followed patients prospectively and assessed the patient’s severity of symptoms, anal incontinence, and quality of life before and after treatment. A member of the direct care team interviewed the patients to collect the data, ensuring objectivity.

This study has several limitations. There is an inherent selection and reporting bias and lack of a comparable group. Furthermore, our sample size is small, and we are not sure whether the improvement in VAS and EQ-5D-5L is clinically important, although it was statistically significant. Indeed, VAS improved to 100 only in 4 patients (23% of the patients who completed the assessment). This might be explained by the inclusion of more severe HD, where 6–8 weeks after the procedure residual symptoms were observed. However, long-term resolution of symptoms after an average follow-up of 20 months was more promising. One patient (4%) confirmed the recurrence of bleeding. Thus, it is possible that RFA treatment offers a good long-term therapeutic benefit.

## Conclusions

RFA for HD is safe, reliable, easy to perform, and is associated with minimal pain and an early return to work/normal activities. Furthermore, we have not seen evidence of functional deterioration or anal incontinence following RFA, but rather significant improvement in patients’ quality of life. Further research to compare RFA to other HD treatment modalities in a randomised controlled trial is the next step to assess the effectiveness and long-term reliability of this procedure.
